# Proteomic Adaptation of *Streptococcus pneumoniae* to the Antimicrobial Peptide Human Beta Defensin 3 (hBD3) in Comparison to Other Cell Surface Stresses

**DOI:** 10.3390/microorganisms8111697

**Published:** 2020-10-30

**Authors:** Pierre-Alexander Mücke, Anne Ostrzinski, Sven Hammerschmidt, Sandra Maaß, Dörte Becher

**Affiliations:** 1 Department of Microbial Proteomics, Institute of Microbiology, Center for Functional Genomics of Microbes, University of Greifswald, Felix-Hausdorff-Str. 8, 17489 Greifswald, Germany; pierre.muecke@uni-greifswald.de (P.-A.M.); anne.ostrzinski@stud.uni-greifswald.de (A.O.); sandra.maass@uni-greifswald.de (S.M.); 2 Department of Molecular Genetics and Infection Biology, Interfaculty Institute for Genetics and Functional Genomics, Center for Functional Genomics of Microbes, University of Greifswald, Felix-Hausdorff-Str. 8, 17489 Greifswald, Germany; sven.hammerschmidt@uni-greifswald.de

**Keywords:** *Streptococcus pneumoniae*, antimicrobial peptides, hBD3, LL-37, adaptation, proteomics

## Abstract

The antimicrobial peptide human Beta defensin 3 (hBD3) is an essential part of the innate immune system and is involved in protection against respiratory pathogens by specifically permeabilizing bacterial membranes. The Gram-positive bacterium *Streptococcus pneumoniae* causes serious diseases including pneumonia, meningitis, and septicemia, despite being frequently exposed to human defense molecules, including hBD3 during colonization and infection. Thus, the question arises how pneumococci adapt to stress caused by antimicrobial peptides. We addressed this subject by analyzing the proteome of *S. pneumoniae* after treatment with hBD3 and compared our data with the proteomic changes induced by LL-37, another crucial antimicrobial peptide present in the human respiratory tract. As antimicrobial peptides usually cause membrane perturbations, the response to the membrane active cationic detergent cetyltrimethylammonium bromide (CTAB) was examined to assess the specificity of the pneumococcal response to antimicrobial peptides. In brief, hBD3 and LL-37 induce a similar response in pneumococci and especially, changes in proteins with annotated transporter and virulence function have been identified. However, LL-37 causes changes in the abundance of cell surface modification proteins that cannot be observed after treatment with hBD3. Interestingly, CTAB induces unique proteomic changes in *S. pneumoniae*. Though, the detergent seems to activate a two-component system that is also activated in response to antimicrobial peptide stress (TCS 05). Overall, our data represent a novel resource on pneumococcal adaptation to specific cell surface stresses on a functional level. This knowledge can potentially be used to develop strategies to circumvent pneumococcal resistance to antimicrobial peptides.

## 1. Introduction

**Figure 1:**
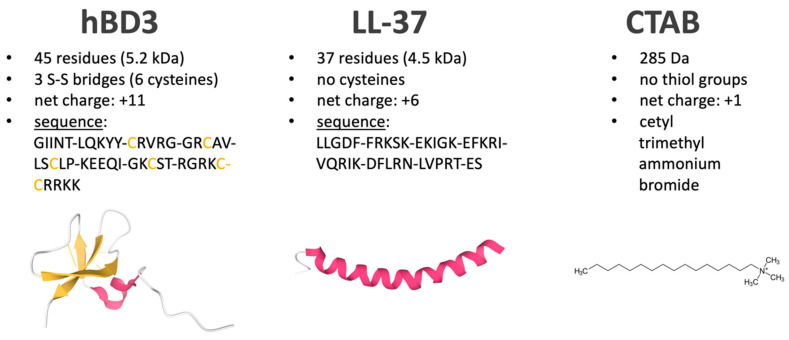
Characteristics of applied stress inducing components hBD3, LL-37, and CTAB. Beta-sheets and cysteines are colored in orange. Alpha-helices are colored in magenta. hBD3 structure (PDB ID: 1KJ6) was obtained from [7] and LL-37 structure (PDB ID: 2K6O) from [8]. Images of structures were created using Mol* [9].

Human Beta defensin 3 (hBD3) is an important member of the class of antimicrobial peptides (AMPs). It is 45 amino acids long (5.2 kDa), has a net positive charge of +11 at pH 7, and a triple stranded β-sheet structure that is stabilized by three disulfide bridges (Figure 1, left) [1]. Two loops of the peptide integrate in bacterial lipid bilayers, interact with negatively charged phospholipids, and finally, permeabilize bacterial membranes leading to leakage of cellular content and cell death [1]. In combination with the α-helical antimicrobial peptide LL-37, hBD3 represents a major constituent of antimicrobial peptides in the respiratory epithelium and is directly involved in innate immunity [2]. Unlike other antimicrobial agents, AMPs are also active against slow growing or dormant cells as they are independent of an active metabolism and instead target the bacterial envelop, making them interesting potential therapeutics [3].

However, exposure to AMPs puts evolutionary pressure on bacteria and therefore, selection for adaptation and resistance is recurrently applied when humans and pathogens interact. In fact, it was shown that bacteria can become resistant to AMPs in culture experiments with increasing peptide concentrations [4,5]. Yet, the functional players causing resistance mainly remained uncovered. Recently, we investigated the proteomic adaptation of *Streptococcus pneumoniae* to the antimicrobial peptide LL-37, a 37 amino acids long peptide (Figure 1, middle) [6]. The data showed that pneumococci may use several adaptation mechanisms to reduce the negative impact of AMPs on their biology, including repulsion, export, and potentially degradation and interception of the peptide.

In this study, we applied our previously established proteomic workflow [6] to investigate the proteomic response of *S. pneumoniae* to hBD3, another major antimicrobial peptide in the human respiratory tract. Furthermore, a basic comparison of LL-37 and hBD3 data allowed us to estimate the specificity of different AMP stresses. Finally, we induced general membrane stress by applying cetyltrimethylammonium bromide (CTAB) (Figure 1, right) to the pneumococcal culture and added the recorded proteomic profiles to our dataset to identify proteomic changes that are uniquely triggered by AMPs.

## 2. Materials and Methods

Cultivation of *Streptococcus pneumoniae* D39 (serotype 2, NCTC 7466), sample preparation for mass spectrometry (MS), MS measurements, and data analysis were performed as described before [6]. Briefly, bacteria were cultivated in modified RPMI 1640 media [10] and stress inducing compounds were applied after 2 h of main culture. To keep the different stresses comparable, we chose a concentration of each compound that reduced the maximal optical density of the culture by around 20%. In a previous study, this was achieved by using 2.5 µg/mL LL-37 acetate (Innovagen, Lund, Sweden) [6]. In the present study, a 20% inhibition was obtained by using 10 µg/mL hBD3 acetate (Innovagen, Lund, Sweden) and 1.25 µg/mL CTAB (Carl Roth, Karlsruhe, Germany), respectively. The current experiments were performed in biological quadruplicates.

Protein samples were prepared for mass spectrometry using S-trap spin columns (Protifi, Huntington, NY, USA) and fractionated using the basic pH reversed-phase peptide fractionation protocol (Thermo Fisher Scientific, Rockford, IL, USA) and self-packed columns. Mass spectrometric measurements were all carried out on an LTQ Orbitrap Velos mass spectrometer in combination with an EASY nLC-1000 liquid chromatography system running 180-min gradients from 1 to 99% acetonitrile containing 0.1% acetic acid. Data quality was monitored by the addition of synthetic iRT peptides (Biognosys, Schlieren, Switzerland). Furthermore, MaxQuant output files and RawMeat (VAST Scientific, Cambridge, MA, USA) were used to detect unexpected changes in MS parameters. As only data passing the quality checks were used in this study, the second replicates of CTAB stress had to be removed from the dataset. MaxQuant [11] versions 1.6.5.0 (CTAB data) and 1.6.10.43 (hBD3 data) were used for protein identification and quantification using parameters as described before [6].

CTAB samples were taken before application of the detergent, after 1 h of stress (and control), and after 2 h of stress (and control). Based on these and previous experiments with LL-37 [6], hBD3 samples were solely taken after 2 h of stress (and control), as proteomic differences between stressed and untreated pneumococci tended to become more distinct at this timepoint. Therefore, analysis was focused on the timepoint following 2 h of stress.

The log_2_(x) transformed data were filtered before in-depth analysis based on the criteria: only identified by site, reverse, and potential contamination using Perseus software [12] version 1.6.5.0 (CTAB data) and 1.6.10.43 (hBD3 data). Additionally, proteins had to be identified in at least 3/4 biological replicates after 2 h of stress or in the control for hBD3 or in at least 2/3 biological replicates under at least 1/5 conditions (before stress, after 1 h or 2 h of stress or in the corresponding control) for CTAB. Annotations were made based on the pneumococcal proteome obtained from Uniprot.org, PneumoBrowse [13], and PSORTdb 3.0 [14]. For statistical testing between the stress and control conditions, Student’s *t*-tests (threshold *p*-value: 0.01) were applied for each independent experiment. Additionally, a minimum fold change of 1.5 was defined in order to emphasize the most prominent proteomic changes. Likewise, on/off proteins were taken into account that were exclusively identified in either stress or control samples.

Finally, all mass spectrometry proteomics data of this study have been deposited to the ProteomeXchange Consortium (http://proteomecentral.proteomexchange.org) via the PRIDE [15] partner repository with the dataset identifier PXD020968.

## 3. Results and Discussion

**Figure 2:**
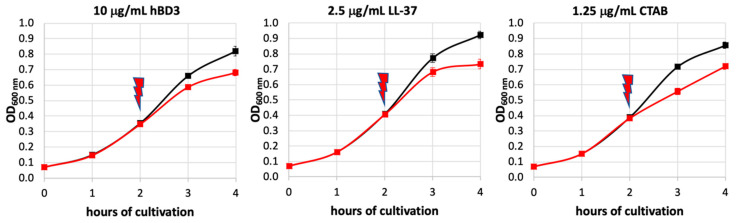
Effect of hBD3, LL-37, or CTAB on the growth of *Streptococcus pneumoniae* D39, respectively. The red arrow indicates the application of either compound after two hours of cultivation. Growth curves obtained under control condition are colored in black. Growth curves obtained under stress condition are colored in red. Error bars represent the standard deviation. *n* = 4 (hBD3), *n* = 6 (LL-37) [6], *n* = 4 (CTAB).

All three applied compounds, hBD3, LL-37 [6], and CTAB, resulted in a concentration-dependent inhibition of bacterial growth. Hence, we selected concentrations of each substance that reduced the optical density of the pneumococcal culture by about 20%. This was achieved by using 10 µg/mL hBD3, 2.5 µg/mL LL-37 [6], and 1.25 µg/mL CTAB, respectively (Figure 2).

A summary of the outcome of the subsequent proteomic analysis is shown in Table 1.

Under all conditions, the pneumococcal proteome, consisting of 1915 proteins, was identified to a high proportion of at least 65%. The proteins, which could be quantified after filtering in Perseus, made up approximately 60% of the annotated proteome. Additionally, each dataset gave rise to normally distributed and covariant data, with high correlation coefficients between the samples of each experiment and between the experiments (at least 0.96, determined by an additional MaxQuant search). Furthermore, the differences between replicates were lower than the differences between the conditions (stress and control) for each experiment (Figures S1 and S2). Using Student’s *t*-tests, the number of significantly changed proteins varied between the experiments. After 2 h of hBD3-induced stress, 60 proteins were changed in abundance (18 were more abundant and 42 less abundant compared to the corresponding control condition). LL-37 caused alterations in 45 proteins (12 were more and 33 less abundant) after 1 h of stress and in 80 proteins (41 more and 39 less abundant) after 2 h of stress [6]. Lastly, CTAB provoked 33 differences (24 more and 9 less abundant proteins) after 1 h of stress and 30 differences with 15 proteins increased and 15 proteins decreased in abundance after 2 h of stress (Table S1). As the general trend was that proteomic differences increased with time, we focused on the timepoint 2 h after stressor application for the following analysis.

Supplementary Table S2 lists all proteins that were significantly changed in at least one experiment following 2 h of stress together with the exact fold changes recorded and the predicted protein localizations and functions. In brief, hBD3 induced several changes in the pneumococcal proteome. The transporter proteins SPD_0686–SPD_0688 were all significantly more abundant in the proteome after hBD3 treatment in comparison to the untreated bacteria. Likewise, the ABC transporter SPD_1214 showed a similar trend, having a fold change (fc) of 3.3 after stressor application. Furthermore, the putative transporter proteins SPD_1525 (fc of 5.7) and SPD_1526 (fc 2.5 without passing our stringent *t*-tests parameters) were enriched after hBD3 stress in combination with their genetic regulator GntR (SPD_1524) (fc of 6.4). On the other hand, SPD_0115 (fc of 0.6), SPD_0161 (fc of 0.4), SPD_1263 (fc of 0.3), SPD_1264 (fc of 0.4), SPD_1267 (only identified under control conditions), and SPD_1514 (fc of 0.2) were significantly less abundant transporters after hBD3 stress. The sortase SrtA (SPD_1076), linking secreted proteins covalently to the peptidoglycan and thus, to the bacterial surface, showed increased levels (fc of 1.6) under hBD3 stress conditions. Interestingly, the two-component system 05 of *S. pneumoniae* seemed to be activated as the sensor histidine kinase CiaH (SPD_0702), the regulator CiaR (SPD_0701) as well as many of their regulated targets [16], the heat-inducible serine protease and chaperone HtrA (SPD_2068), the metabolic protein MalP (SPD_1932), the foldase PrsA (SPD_0868), and the protein of unknown function SPD_0913 were all significantly more abundant following hBD3 treatment. Additionally, the genetic neighbor of MalP (SPD_1932), MalQ (SPD_1933), was enriched with a moderate (non-significant) fold change of 1.5. Then, the proteins with unknown function SPD_1515 (just slightly decreased in abundance) and SPD_1516–SPD_1517 (severely depleted with a fold change of 0.1 and 0.2, respectively) were altered after hBD3 application. Lastly, the histidine triad virulence proteins PhpA (SPD_1038), PhtD (SPD_0889), and PhtE (SPD_0890) were all significantly less abundant after stress induced by hBD3.

**Figure 3:**
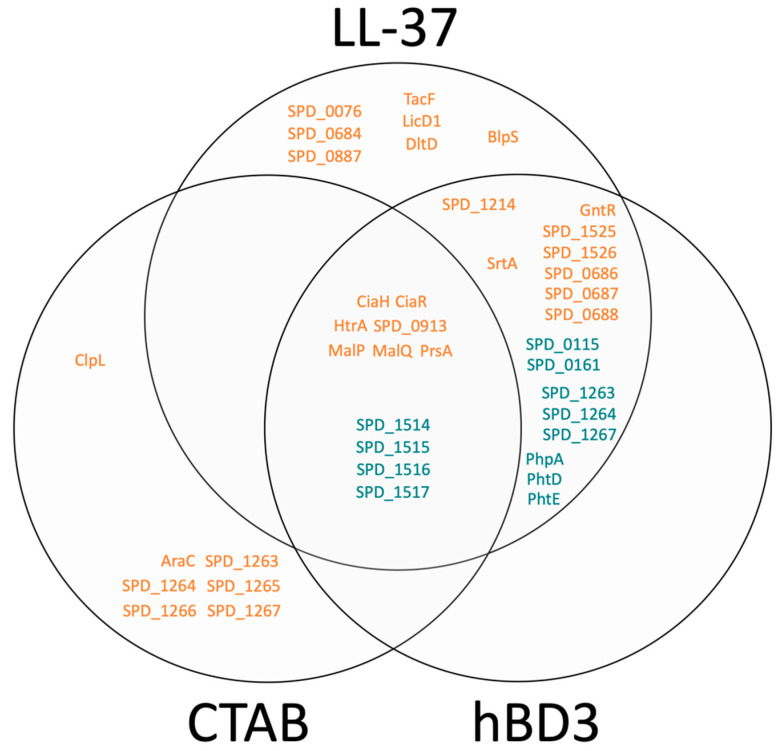
Comparison between most prominent changes in protein abundance in response to LL-37, hBD3, or CTAB stress, respectively. Colors indicate if a protein was more (orange) or less (turquoise) abundant after application of the corresponding stressor in comparison to the experiment’s control condition.

In order to compare the proteomic response of *S. pneumoniae* to hBD3 with proteomic responses to other antimicrobial peptides, such as LL-37, the results obtained in this study were compared to those recently published [6]. In principle, the pneumococcal responses to hBD3 and LL-37 appear very similar (Figure 3).

The mentioned transporters, virulence proteins, the sortase SrtA, the genetic regulator GntR, and the two-component system 05 (CiaHR) including its targets (HtrA, MalP, MalQ, PrsA, SPD_0913), and the proteins of unknown function SPD_1515–SPD_1517 were all changed in a similar way. Interestingly, the crystal structure of the transporter proteins SPD_0686–SPD_0688, which were more abundant after hBD3 and LL-37 stress, were solved recently, and knockout mutation of the corresponding genes increased the pneumococcal sensitivity towards LL-37 [17]. Taken together, the findings suggest that SPD_0686–SPD_0688 function as an inducible channel that directs AMPs from the bacterial membrane to the peptidoglycan layer and thus, decreases the detrimental interaction between the AMP and the membrane [17]. However, in contrast to the proteomic changes induced by hBD3, LL-37 stress caused a significant increase in BlpS, the transporters SPD_0076, SPD_0684, and SPD_0887, and the cell surface modification proteins TacF (SPD_1128), LicD1 (SPD_1129), and DltD (SPD_2002). The changes in cell surface modification proteins are thought to decrease the negative surface charge of pneumococci and therefore, reduce the attraction and binding of positively charged antibacterial LL-37 to the bacterial surface [18,19,20,21,22]. Supporting the role of the *dlt* locus in AMP resistance, a *dltD* null mutation was shown to increase the susceptibility of *S. pneumoniae* to LL-37 [6] and the gene segment was found to be under strong selective pressure in repeated murine nasal colonization with pneumococci [23]. In detail, null mutation of *dltB* resulted in an increased adherence to lung epithelial cells and the absence of *dltB* enhanced pneumococcal fitness in nasal colonization with serotypes 19F and 2 but not with the more positively charged serotypes 7F and 15B. On the other side, *dltB* mutation resulted in an enhanced sensitivity to the antimicrobial peptide LL-37 and reduced fitness in lower lung infections. This indicates that the outcome of potential cell surface modifications on pneumococcal fitness are niche and capsule specific. As our data also show significant changes in abundance of a protein encoded by the *dlt* locus (DltD) in response to LL-37 but not in response to hBD3 treatment, the potential adaptations in the pneumococcal cell surface may additionally be dependent on the peptide type.

In contrast to the relatively high correlation between proteomic changes in the pneumococcus seen after treatment with either hBD3 or LL-37 (except from changes in abundance of cell surface proteins), the modification of the bacterial proteome in response to general membrane stress induced by the cationic detergent CTAB was rather different (Figure 3). No significant changes in cell surface modification proteins, virulence proteins, the GntR regulator, many transporters under control of GntR (SPD_0686–SPD_0688 and SPD_1525–SPD_1526) and the transporters SPD_1214, SPD_0115, or SPD_0161 could be detected. Additionally, the transporter proteins and AraC family regulator SPD_1262–SPD_1267 were all significantly more abundant after CTAB stress (mainly with fold changes of over 10), whereas these proteins were not changed or even (significantly) less abundant in response to hBD3 and LL-37, respectively. Moreover, the Clp protease subunit ClpL (SPD_0308) showed a 7-fold change after CTAB treatment but was not affected by any of the examined AMPs. Interestingly, the two-component system 05 also appeared to be activated by CTAB. Similar to the proteomic changes induced by hBD3 and LL-37, respectively, CiaH, CiaR, and the aforementioned targets of this regulatory system were all more abundant after CTAB stress, even when the detected protein fold changes were less pronounced compared to the changes induced by the two antimicrobial peptides. Hence, general membrane stress triggered by the detergent CTAB affected the same regulatory system as the antimicrobial peptides hBD3 and LL-37 in our in vitro experimental setup. Additionally, the unknown function proteins SPD_1515–SPD_1517 and the transporter SPD_1514 were, as in response to AMP treatment, less abundant after CTAB application. This effect can possibly be explained by a general attraction of positively charged molecules (cationic detergent CTAB and AMPs) by these putative membrane proteins.

## 4. Conclusion

To conclude, *S. pneumoniae* reacts to antimicrobial peptides by proteomic adaptations that likely decrease the antimicrobial effect of those peptides. The adaptations include changes in protein abundance of exporters that potentially guide away AMPs from the bacterial surface [24], a protease that potentially deactivates AMPs as shown for several other proteases and bacteria [25], and surface-exposed virulence factors that may reduce sensing by the immune system [26]. In the case of LL-37, severe adaptations in proteins were annotated as surface modification proteins could be observed 2 h after application of the compound [6]. In fact, the enriched proteins are expected to reduce the bacterial surface charge and thus, the attraction between pneumococci and the cationic peptide. On the other side, general membrane stress induced by detergents and stress induced by AMPs activate the same two-component system (TCS 05). Hence, this response is rather unspecific to human immune molecules. However, it cannot be excluded to play a role in pneumococcal adaptation to AMPs.

To prevent the potential risk of selecting pneumococcal strains resistant to human immune molecules by directly applying AMPs as drugs [4,27], it should be considered to rather block potential pneumococcal adaptation mechanisms. This could be achieved unspecifically by interrupting the CiaHR system, LL-37 specifically by preventing changes in cell surface modification proteins, or AMP specifically by blocking specific changes in transporter abundances. Another strategy is the use of AMP-derived molecules with different structures for medical treatment, optimally modified with non-canonical amino acids to protect the agent from proteolytic degradation [28,29]. These analogues, like natural AMPs, often act synergistically with classical antibiotics by paving the way for intracellular antibiotic targets [28,30]. Additionally, cross-resistance of classical antibiotics and antimicrobial peptides was hardly observed [31,32]. Ultimately, AMP resistance would solely affect direct AMP killing of bacteria and not the immunomodulatory properties of the peptides [33] or other parts of the innate immune system like phagocytic killing, complement-dependent killing, or adaptive immunity [34].

## Figures and Tables

**Table 1:** Summary of proteomic analyses. Arrows indicate the number of proteins that were statistically more or less abundant after two hours of stressor application in comparison to the experiment’s control condition. On/off proteins are included. * Student’s *t*-test (*p*-value = 0.01, min. fold change= 1.5). On/off proteins are proteins which were exclusively identified in either stress or control samples. Data for LL-37 treatment were obtained from [6]. *n* = 4 (hBD3), *n* = 6 (LL-37) [6], *n* = 3 (CTAB).

Applied Compound	Identified Proteins (% of the Total Proteome)	Quantifiable Proteins (% of the Total Proteome)	Pearson Correlation between Samples of the Experiment	Number of Proteins with Significantly * Changed Abundance after 2 h of Stress Including on/off Proteins
hBD3	1241 (65%)	1106 (58%)	0.98–0.99	60 (18↑, 42↓)
LL-37 [6]	1293 (68%)	1118 (58%)	0.96–0.99	80 (41↑, 39↓)
CTAB	1275 (67%)	1184 (62%)	0.96–0.99	30 (15↑, 15↓)

## Supplementary Materials

The following are available online at https://www.mdpi.com/2076-2607/8/11/1697/s1, Figure S1: Scatter plots of hBD3 samples; Figure S2: Scatter plots of CTAB samples; Table S1: Significant changes in protein abundance of *S. pneumoniae* D39 upon CTAB exposure; Table S2: Changes in protein abundance of *S. pneumoniae* D39 upon hBD3, LL-37, or CTAB exposure, respectively.
