# Supplementary material for: Proteomic Adaptation of Streptococcus pneumoniae to the Antimicrobial Peptide Human Beta Defensin 3 (hBD3) in Comparison to Other Cell Surface Stresses
Source: Microorganisms. 2020 Oct 30;8(11):1697. doi: 10.3390/microorganisms8111697 (PMC7694020; doi:10.3390/microorganisms8111697)
Supplement: Supplementary file 1 [file microorganisms-08-01697-s001.zip › 201023_Supplementals/200901_Table_S1.pdf]

Table S1: Significant changes in protein abundance of *S. pneumoniae* D39 upon CTAB exposure

Significant* changes in protein abundance are shown in bold. On/off proteins are proteins that are exclusively identified in either stress or control samples. Arrows indicate if a protein was more or less abundant after application of CTAB in comparison to the control condition. “nc”: fold change not calculable because of low identification rate. * Student’s *t*-test (*p*-value= 0.01, min. fold change= 1.5).

CTAB stress ↑

locusID	protein	localization<br>PSORTdb_3.0 [1]	protein fold change<br>1 h stress	 2 h stress
SPD_0091	Unknown	Cytoplasmic	1.3	**1.7**
SPD_0093	Putative membrane protein PtvC	Cytoplasmic Membrane	**1.6**	1.0
SPD_0094	Putative membrane-associated protein PvtB	Unknown	**1.8**	1.1
SPD_0166	Lumazine synthase RibH	Cytoplasmic	**3.5**	1.6
SPD_0167	Riboflavin biosynthesis protein RibB	Cytoplasmic	**2.9**	1.4
SPD_0169	Riboflavin biosynthesis protein RibD	Cytoplasmic	**2.6**	1.9
SPD_0184	Putative lipoprotein	Extracellular	**2.0**	1.3
SPD_0240	Acetyltransferase	Unknown	3.9	**3.0**
SPD_0265	Alcohol dehydrogenase AdhA	Cytoplasmic	**2.3**	**1.7**
SPD_0308	ATP-dependent Clp protease, subunit ClpL	Cytoplasmic	**14.4**	**7.0**
SPD_0351	Sensor histidine kinase LiaS	Cytoplasmic Membrane	**1.7**	2.0
SPD_0372	ABC transporter	Cytoplasmic Membrane	**1.6**	1.7
SPD_0420	Pyruvate formate lyase PflB	Cytoplasmic	**1.7**	1.1
SPD_0646	Unknown	Cytoplasmic	**1.6**	1.4
SPD_0701	Response regulator CiaR	Cytoplasmic	**1.9**	1.4
SPD_0803	Unknown	Cytoplasmic Membrane	**on**	**on**
SPD_0868	Foldase PrsA	Cytoplasmic Membrane	**1.8**	**2.1**
SPD_0913	Unknown	Unknown	**1.9**	**2.0**
SPD_1262	Transcriptional regulator AraC family	Cytoplasmic	1.2	**2.0**

SPD_1263	ABC transporter	Cytoplasmic Membrane	**8.5**	**11.2**
SPD_1264	ABC transporter	Cytoplasmic Membrane	**7.4**	**11.8**
SPD_1265	Putative membrane protein	Cytoplasmic Membrane	**on**	**on**
SPD_1266	Putative membrane protein	Cytoplasmic Membrane	nc	**on**
SPD_1267	ABC transporter	Cytoplasmic Membrane	**6.9**	**19.9**
SPD_1793	Universal stress protein	Cytoplasmic	**1.6**	0.8
SPD_1834	Aldehyde-alcohol dehydrogenase	Cytoplasmic	**2.0**	1.2
SPD_1932	Alpha-1,4 glucan phosphorylase MalP	Cytoplasmic Membrane	**1.9**	**1.8**
SPD_2068	Serine protease HtrA	Extracellular	**3.1**	**2.6**

CTAB stress ↓

**locusID**	**protein**	**localization**	**protein fold change**	
		PSORTdb_3.0 [1]	1 h stress	2 h stress
SPD_0095	Putative integral membrane protein PtvA	Cytoplasmic Membrane	1.0	**0.4**
SPD_0104	LysM domain protein	Cytoplasmic Membrane	**0.5**	**0.3**
SPD_0321	Glycosyl transferase Cps2F	Cytoplasmic Membrane	**0.3**	**0.4**
SPD_0558	Serine protease PrtA	Cellwall	**0.6**	0.9
SPD_0736	Pyrimidine-nucleoside phosphorylase Pdp	Cytoplasmic	1.0	**0.6**
SPD_0960	Glycosyl transferase CpoA	Cytoplasmic	**0.6**	0.6
SPD_1153	Phosphoesterase Pde2	Cytoplasmic	**0.7**	1.0
SPD_1211	3-dehydroquinate dehydratase I AroD	Cytoplasmic	0.9	**0.6**
SPD_1246	Glucosamine-6-phosphate deaminase NagB	Cytoplasmic	0.8	**0.6**
SPD_1250	NAD synthetase NadE	Cytoplasmic	0.9	**0.6**
SPD_1428	Cytidylate kinase Cmk	Cytoplasmic	0.9	**0.7**
SPD_1449	Unknown	Cytoplasmic	0.9	**0.5**
SPD_1514	ABC transporter	Cytoplasmic Membrane	0.5	**0.2**

SPD_1516	Putative membrane protein	Cytoplasmic Membrane	0.5	**0.3**
SPD_1527	Putative sodium ABC exporter	Cytoplasmic Membrane	**0.7**	**0.6**
SPD_1597	Tryptophan synthase beta chain TrpB	Cytoplasmic	0.7	**0.6**
SPD_1600	Anthranilate phosphoribosyltransferase TrpD	Cytoplasmic	0.7	**0.5**
SPD_1601	Anthranilate synthase component II TrpG	Cytoplasmic	**0.5**	0.5
SPD_1634	Galactokinase GalK	Cytoplasmic	**off**	nc
SPD_1928	Unknown	Unknown	**0.6**	**0.4**

References

1. Peabody, M.A.; Laird, M.R.; Vlasschaert, C.; Lo, R.; Brinkman, F.S. PSORTdb: expanding the bacteria and archaea protein subcellular localization database to better reflect diversity in cell envelope structures. *Nucleic Acids Res.* **2016**, *44*, D663-D668, doi:10.1093/nar/gkv1271.
