# Supplementary material for: Proteomic Adaptation of Streptococcus pneumoniae to the Antimicrobial Peptide Human Beta Defensin 3 (hBD3) in Comparison to Other Cell Surface Stresses
Source: Microorganisms. 2020 Oct 30;8(11):1697. doi: 10.3390/microorganisms8111697 (PMC7694020; doi:10.3390/microorganisms8111697)
Supplement: Supplementary file 1 [file microorganisms-08-01697-s001.zip › 201023_Supplementals/200901_Table_S2.pdf]

## Table S2: Changes in protein abundance of *S. pneumoniae* D39 upon hBD3, LL-37, or CTAB exposure, respectively

Significant* changes in protein abundance are shown in bold. On/off proteins are proteins that are exclusively identified in either stress or control samples. Arrows indicate if a protein was more or less abundant after application of the corresponding stressor in comparison to the experiment's control condition. "nd": not detected. "nc": fold change not calculable because of low identification rate. * Student's *t*-test (*p*-value= 0.01, min. fold change= 1.5).

| **locusID** | **protein** | **localization** PSORTdb_3.0 [1] | **protein fold change** (after 2 hours of stress) | | |
|---|---|---|---|---|---|
| | | | hBD3 | LL-37 [2] | CTAB |
| **transporters ↑** | | | | | |
| SPD_0076 | Potassium uptake protein | Cytoplasmic Membrane | 1.2 | **2.9** | 0.6↓ |
| SPD_0107 | Putative bacteriocin immunity protein | Cytoplasmic Membrane | 1.2 | **1.8** | 0.9↓ |
| SPD_0372 | ABC transporter | Cytoplasmic Membrane | **1.9** | 2 | 1.7 |
| SPD_0464 | ABC transporter EcsA | Cytoplasmic Membrane | 0.9↓ | **1.6** | 1.1 |
| SPD_0684 | Biotin transporter | Cytoplasmic Membrane | 0.5↓ | **4.3** | 0.7↓ |
| SPD_0686 | Efflux transporter | Unknown | **1.7** | 1.5 | 1.0 |
| SPD_0687 | ABC transporter | Cytoplasmic Membrane | **1.7** | **1.6** | 1.1 |
| SPD_0688 | Efflux ABC transporter | Cytoplasmic Membrane | **1.6** | **2.1** | 1.0 |
| SPD_0861 | Protein-export membrane protein SecG | Cytoplasmic Membrane | nd | **2.2** | 0.5↓ |
| SPD_0887 | Cationic amino acid transporter | Cytoplasmic Membrane | 1.7 | **5.4** | 1.7 |
| SPD_1169 | Oligopeptide ABC transporter | Cytoplasmic Membrane | 1.4 | **2.8** | 0.6↓ |
| SPD_1214 | ABC transporter | Cytoplasmic Membrane | 3.3 | **4.7** | 1.3 |
| SPD_1525 | Multidrug ABC transporter | Cytoplasmic Membrane | **5.7** | **7.5** | 1.2 |
| SPD_1526 | Putative ABC transporter | Cytoplasmic Membrane | 2.5 | **on** | nd |
| SPD_2024 | Thiamin ABC transporter | Cytoplasmic Membrane | 0.8↓ | **2.9** | 0.6↓ |
| **transporters ↓** | | | | | |
| SPD_0115 | Putative efflux transporter | Cytoplasmic Membrane | **0.6** | **0.5** | 1.0 |
| SPD_0161 | Putative divalent metal cation transporter | Cytoplasmic Membrane | **0.4** | **0.1** | 1.0 |
| SPD_0374 | Putative ABC transporter | Cytoplasmic Membrane | nd | **off** | nd |

| | | | | | |
|---|---|---|---|---|---|
| SPD_0634 | Copper-exporting ATPase | Cytoplasmic | **0.4** | 0.7 | 0.5 |
| SPD_0635 | Cation-transporting ATPase | Cytoplasmic Membrane | **0.5** | **0.6** | 0.8 |
| SPD_1176 | ABC transporter | Cytoplasmic Membrane | 0.9 | **0.6** | 0.7 |
| SPD_1263 | ABC transporter | Cytoplasmic Membrane | **0.3** | **0.6** | **11.2↑** |
| SPD_1264 | ABC transporter | Cytoplasmic Membrane | **0.4** | 0.7 | **11.8↑** |
| SPD_1265 | Putative membrane protein | Cytoplasmic Membrane | nd | nc | **on↑** |
| SPD_1266 | Putative membrane protein | Cytoplasmic Membrane | nd | nd | **on↑** |
| SPD_1267 | ABC transporter | Cytoplasmic Membrane | **off** | 0.7 | **19.9↑** |
| SPD_1463 | ABC transporter | Cytoplasmic Membrane | **0.6** | 0.7 | 0.9 |
| SPD_1466 | ABC transporter | Cytoplasmic Membrane | 0.8 | **0.7** | 1.0 |
| SPD_1514 | ABC transporter | Cytoplasmic Membrane | **0.2** | **0.2** | **0.2** |
| SPD_1527 | Putative sodium ABC exporter | Cytoplasmic Membrane | **0.3** | **0.3** | **0.6** |
| SPD_1838 | Preprotein translocase YajC subunit | Unknown | 1.1↑ | **0.6** | 1.1↑ |
| SPD_1997 | Zinc ABC transporter AdcA | Cytoplasmic Membrane | **0.7** | **0.6** | 0.7 |

**cell surface modification ↑**

| | | | | | |
|---|---|---|---|---|---|
| SPD_0099 | Capsular polysaccharide biosynthesis protein CapD | Cytoplasmic Membrane | 1.4 | **1.5** | 1.3 |
| SPD_0873 | Putative lyzozyme | Cytoplasmic | 1.2 | **1.6** | 0.6↓ |
| SPD_1076 | Sortase SrtA | Cytoplasmic Membrane | **1.6** | **1.6** | 1.3 |
| SPD_1128 | Putative teichoic acid flippase TacF | Cytoplasmic Membrane | 0.7 | **3.5** | 2.6 |
| SPD_1129 | Phosphotransferase LicD1 | Cytoplasmic | 0.9↓ | **1.8** | 0.7↓ |
| SPD_2002 | DltD | Cytoplasmic Membrane | 1.0 | **1.7** | 1.3 |

**cell surface modification ↓**

| | | | | | |
|---|---|---|---|---|---|
| SPD_0307 | Phospho-N-acetylmuramoyl-pentapeptide-transferase | Cytoplasmic Membrane | **0.7** | 0.8 | 0.3 |
| SPD_0321 | Glycosyl transferase Cps2F | Cytoplasmic Membrane | 0.9 | 0.8 | **0.4** |

**protease ↑**

| | | | | | |
|---|---|---|---|---|---|
| SPD_2068 | Serine protease HtrA | Extracellular | **2.7** | **2.0** | **2.6** |
| SPD_0308 | ATP-dependent Clp protease, subunit ClpL | Cytoplasmic | 0.8↓ | 1.0 | **7.0** |
| **metabolism ↑** | | | | | |
| SPD_1932 | Alpha-1,4 glucan phosphorylase MalP | Cytoplasmic Membrane | **1.9** | **2.2** | **1.8** |
| SPD_1933 | 4-alpha-glucanotransferase MalQ | Cytoplasmic | 1.5 | **2.3** | 1.9 |
| SPD_1664 | Sugar PTS system | Cytoplasmic Membrane | 0.8↓ | **1.8** | 1.8 |
| **metabolism ↓** | | | | | |
| SPD_0070 | Sugar isomerase domain protein AgaS | Cytoplasmic | **off** | nc | 0.6 |
| SPD_0736 | Pyrimidine-nucleoside phosphorylase Pdp | Cytoplasmic | 0.7 | **0.6** | **0.6** |
| SPD_0895 | Ferrochelatase HemH | Cytoplasmic | nd | **0.3** | 0.5 |
| SPD_1078 | L-lactate dehydrogenase L-LDH | Cytoplasmic | 1.2↑ | **0.7** | 1.0 |
| SPD_1211 | 3-dehydroquinate dehydratase I AroD | Cytoplasmic | 0.9 | 0.9 | **0.6** |
| SPD_1246 | Glucosamine-6-phosphate deaminase NagB | Cytoplasmic | **0.6** | **0.6** | **0.6** |
| SPD_1250 | NAD synthetase NadE | Cytoplasmic | 0.8 | 0.7 | **0.6** |
| SPD_1428 | Cytidylate kinase Cmk | Cytoplasmic | 0.9 | 0.6 | **0.7** |
| SPD_1597 | Tryptophan synthase beta chain TrpB | Cytoplasmic | 0.8 | 0.8 | **0.6** |
| SPD_1600 | Anthranilate phosphoribosyltransferase TrpD | Cytoplasmic | 0.7 | 0.7 | **0.5** |
| SPD_1832 | Sugar PTS system | Cytoplasmic | **off** | nc | 0.5 |
| SPD_1834 | Aldehyde-alcohol dehydrogenase | Cytoplasmic | 0.7 | **0.7** | 1.2↑ |
| SPD_1934 | Maltose ABC transporter MalX | Cytoplasmic Membrane | 0.8 | **0.6** | 0.9 |
| **virulence ↓** | | | | | |
| SPD_0126 | Pneumococcal surface protein A | Extracellular | **0.6** | 0.8 | 1.0 |
| SPD_0579 | Choline binding protein CbpL | Extracellular | **0.6** | 0.8 | 1.0 |
| SPD_0889 | Pneumococcal histidine triad protein D PhtD | Extracellular | **0.6** | 0.7 | 0.9 |
| SPD_0890 | Pneumococcal histidine triad protein E PhtE | Extracellular | **0.5** | **0.6** | 1.0 |

| | | | | | |
|---|---|---|---|---|---|
| SPD_1038 | Pneumococcal histidine triad protein A PhpA | Extracellular | **0.5** | **0.6** | 1.0 |
| SPD_1965 | Choline binding protein PcpA | Extracellular | **0.5** | 0.8 | 0.7 |
| SPD_2017 | Choline binding protein A (CbpA/PspC) | Extracellular | **0.6** | 0.9 | 0.9 |
| | | | | | |
| **other ↑** | | | | | |
| SPD_0170 | Holliday junction ATP-dependent DNA helicase RuvA | Cytoplasmic | 1.5 | **1.5** | 1.1 |
| SPD_0240 | Acetyltransferase | Unknown | 0.9↓ | 0.5↓ | **3.0** |
| SPD_0265 | Alcohol dehydrogenase AdhA | Cytoplasmic | 1.2 | 1.1 | **1.7** |
| SPD_0476 | Phosphotransferase enzyme family protein | Cytoplasmic | 1.0 | **1.6** | 0.8↓ |
| SPD_0534 | Tributyrin esterase EstA | Unknown | 1.2 | **1.5** | 1.2 |
| SPD_0863 | SsrA-binding protein (Small protein B) SmpB | Cytoplasmic | 1.4 | **1.5** | 1.0 |
| SPD_0868 | Foldase PrsA | Cytoplasmic Membrane | **1.8** | **1.6** | **2.1** |
| SPD_0957 | DNA primase DnaG | Cytoplasmic | **1.5** | **1.7** | 1.5 |
| SPD_0989 | 50S ribosomal protein L21 | Unknown | **1.5** | 1.2 | 1.2 |
| SPD_1023 | Tyrosine recombinase XerS | Cytoplasmic | 0.8↓ | **1.7** | 0.9↓ |
| SPD_1240 | Heme chaperone HemW | Cytoplasmic | **1.6** | 1.5 | 0.7↓ |
| SPD_1424 | tRNA pseudouridine synthase A TruA | Cytoplasmic | 1.1 | **2.8** | 1.4 |
| SPD_1439 | 30S ribosomal protein S15 RpsO | Cytoplasmic | 1.4 | **1.5** | 1.2 |
| SPD_1546 | Primosomal protein PriA | Cytoplasmic | 1.5 | **1.8** | 1.1 |
| SPD_1657 | Tyrosine recombinase XerD-like | Cytoplasmic | nc | **2.1** | 1.4 |
| SPD_1822 | Ribosomal large subunit pseudouridine synthase | Cytoplasmic | 1.4 | **1.9** | 1.0 |
| SPD_1963 | 50S ribosomal protein L32 | Cytoplasmic | **2.2** | nd | 1.0 |
| | | | | | |
| **other ↓** | | | | | |
| SPD_0008 | Cell division protein DivIC | Cytoplasmic Membrane | **off** | 0.8 | 0.9 |
| SPD_0128 | MutT/nudix family protein | Cytoplasmic | 1.0 | **0.5** | 1.3↑ |
| SPD_0305 | Cell division protein FtsL | Cytoplasmic | **off** | nc | 1.0 |
| SPD_0886 | Thioredoxin family protein Etrx2 | Extracellular | **0.5** | **0.5** | 0.5 |

| | | | | | |
|---|---|---|---|---|---|
| SPD_1260 | Methyltransferase | Cytoplasmic | 0.7 | **0.7** | 0.8 |
| SPD_1280 | Cof family protein | Cytoplasmic | 0.7 | **0.7** | 0.7 |
| SPD_1301 | NADPH-dependent FMN reductase | Cytoplasmic | 0.5 | **0.6** | 0.6 |
| SPD_1302 | Putative oxidoreductase | Unknown | **0.6** | **0.4** | 0.6 |
| SPD_1375 | Putative NADPH-dependent FMN reductase | Unknown | **0.5** | **0.6** | 1.1↑ |
| SPD_1397 | Endoribonuclease L-PSP | Cytoplasmic | **0.4** | 0.9 | 0.6 |
| SPD_1464 | Thioredoxin peroxidase Tpx PsaD | Unknown | 0.7 | **0.6** | 0.8 |
| SPD_1477 | Cell division protein SepF | Cytoplasmic | 0.9 | **0.6** | 0.7 |
| SPD_1520 | Nitroreductase | Unknown | **0.6** | 0.7 | 0.8 |
| SPD_1590 | Putative general stress protein 24 | Cytoplasmic | **0.6** | **0.6** | 0.7 |
| SPD_1964 | 50S ribosomal protein L33 | Cytoplasmic | **0.5** | 1.2↑ | 1.9 |
| SPD_2043 | Secreted 45 kDa protein PcsB | Extracellular | 1.0 | **0.7** | 0.7 |
| **gene regulation ↑** | | | | | |
| SPD_0467 | BlpS | Cytoplasmic | nc | **1.8** | nc |
| SPD_0701 | Response regulator CiaR | Cytoplasmic | **1.9** | **1.6** | 1.4 |
| SPD_0702 | Sensor histidine kinase CiaH | Cytoplasmic Membrane | **3.3** | 2.1 | 1.6 |
| SPD_1262 | Transcriptional regulator AraC family | Cytoplasmic | 0.8↓ | 1.0 | **2.0** |
| SPD_1366 | Putative general stress protein 13 | Cytoplasmic | 0.9↓ | **2.2** | 1.2 |
| SPD_1524 | Transcriptional regulator GntR | Cytoplasmic | **6.4** | **8.3** | 1.9 |
| SPD_2063 | Response regulator ComE | Cytoplasmic | 1.4 | **1.5** | 1.3 |
| **gene regulation ↓** | | | | | |
| SPD_0309 | Autoinducer-2 production protein LuxS | Cytoplasmic | 0.8 | **0.5** | 0.9 |
| SPD_0525 | Sensor histidine kinase VncS | Cytoplasmic | **off** | 1.0 | 0.7 |
| SPD_0633 | Putative transcriptional regulator CopY | Cytoplasmic | 0.6 | **0.5** | 0.6 |
| SPD_1904 | Arginine repressor ArgR | Cytoplasmic | 1.0 | **0.7** | 1.0 |

| | | | | | |
|---|---|---|---|---|---|
| **unknown ↑** | | | | | |
| SPD_0091 | Unknown | Cytoplasmic | 1.2 | 1.1 | **1.7** |
| SPD_0803 | Unknown | Cytoplasmic Membrane | nc | nc | **on** |
| SPD_0913 | Unknown | Unknown | **2.9** | **2.8** | **2.0** |
| SPD_1506 | Unknown | Cytoplasmic | **2.4** | nc | 1.6 |
| **unknown ↓** | | | | | |
| SPD_0039 | Unknown | Cytoplasmic | 0.8 | **0.6** | 0.8 |
| SPD_0095 | Putative integral membrane protein PtvA | Cytoplasmic Membrane | 0.8 | nc | **0.4** |
| SPD_0104 | LysM domain protein | Cytoplasmic Membrane | 0.8 | 0.7 | **0.3** |
| SPD_0145 | Unknown | Unknown | **off** | nc | 0.6 |
| SPD_0714 | Unknown | Cytoplasmic | **0.5** | **0.6** | 0.7 |
| SPD_0792 | Putative lipoprotein | Unknown | 0.9 | **0.6** | 0.9 |
| SPD_0816 | Unknown | Cytoplasmic | **0.6** | 1.0 | 0.7 |
| SPD_1102 | Unknown | Cytoplasmic | **0.5** | 0.8 | 0.7 |
| SPD_1103 | Unknown | Cytoplasmic | **0.6** | 0.9 | 0.8 |
| SPD_1449 | Unknown | Cytoplasmic | 1.0 | 0.8 | **0.5** |
| SPD_1515 | Putative membrane protein | Cytoplasmic Membrane | nc | **0.4** | 0.3 |
| SPD_1516 | Putative membrane protein | Cytoplasmic Membrane | **0.1** | **0.2** | **0.3** |
| SPD_1517 | Unknown | Unknown | **0.2** | **0.3** | 0.4 |
| SPD_1588 | Unknown | Cytoplasmic Membrane | **0.5** | 0.7 | 0.7 |
| SPD_1644 | Unknown | Cytoplasmic | **0.6** | 1.1↑ | 0.9 |
| SPD_1928 | Unknown | Unknown | 0.8 | 0.7 | **0.4** |
| SPD_2029 | Unknown | Cytoplasmic | **off** | 1.3↑ | 0.9 |

**References**

1. Peabody, M.A.; Laird, M.R.; Vlasschaert, C.; Lo, R.; Brinkman, F.S. PSORTdb: expanding the bacteria and archaea protein subcellular localization database to better reflect diversity in cell envelope structures. *Nucleic Acids Res.* **2016**, *44*, D663-D668, doi:10.1093/nar/gkv1271.
2. Mücke, P.-A.; Maaß, S.; Kohler, T.P.; Hammerschmidt, S.; Becher, D. Proteomic adaptation of *Streptococcus pneumoniae* to the human antimicrobial peptide LL-37. *Microorganisms* **2020**, *8*, doi:10.3390/microorganisms8030413.
