# Supplementary material for: Proteomic Adaptation of Streptococcus pneumoniae to the Antimicrobial Peptide Human Beta Defensin 3 (hBD3) in Comparison to Other Cell Surface Stresses
Source: Microorganisms. 2020 Oct 30;8(11):1697. doi: 10.3390/microorganisms8111697 (PMC7694020; doi:10.3390/microorganisms8111697)
Supplement: Supplementary file 1 [file microorganisms-08-01697-s001.zip › 201023_Supplementals/201023_Figure_S1-S2.pdf]

Figure S1: Scatter plots of hBD3 samples

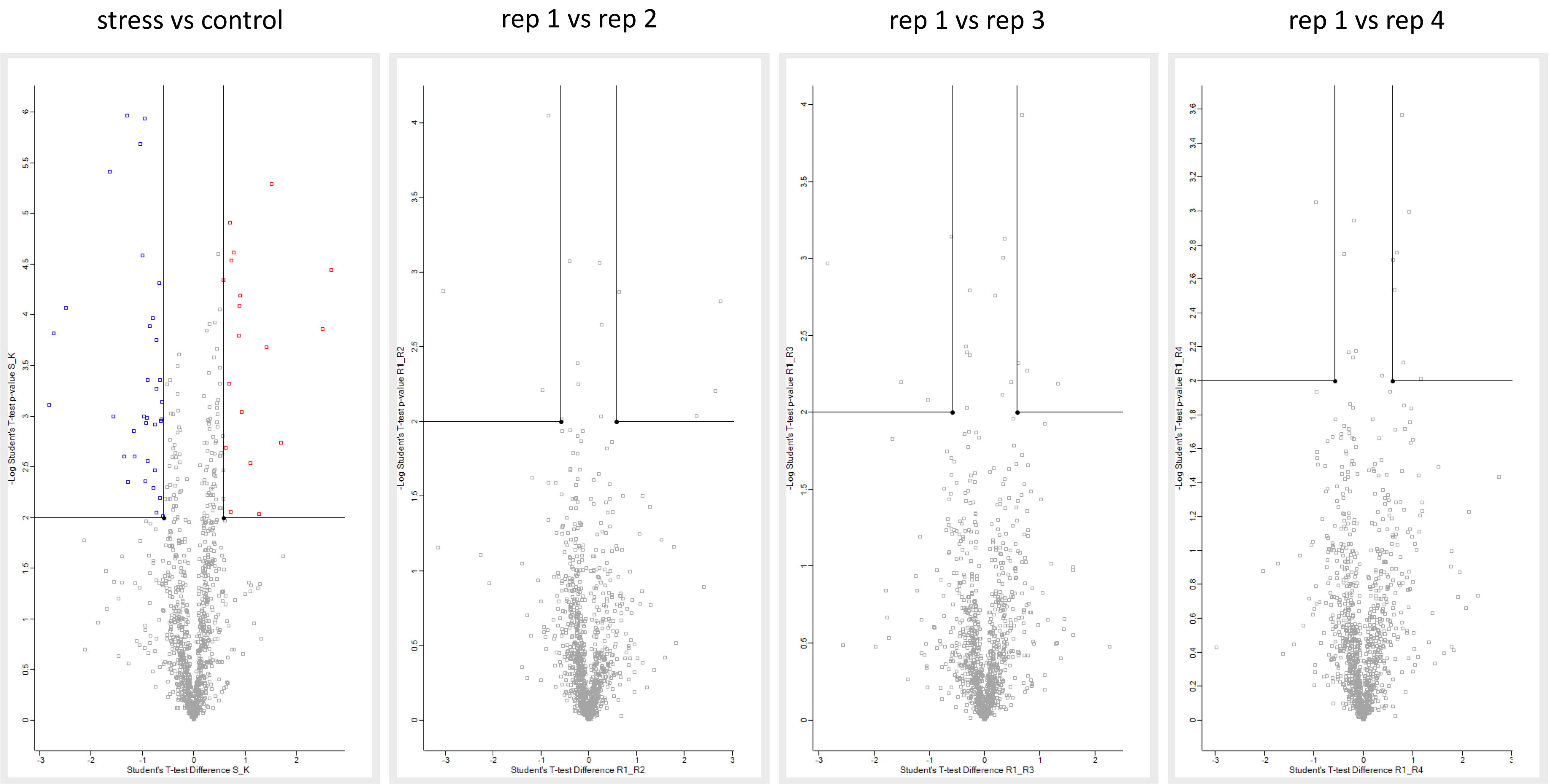

Note that on/off proteins are not shown as significantly changed proteins as no valid fold change and p-value can be assigned to them.

Figure S1: Scatter plots of hBD3 samples (continued)

rep 2 vs rep 3

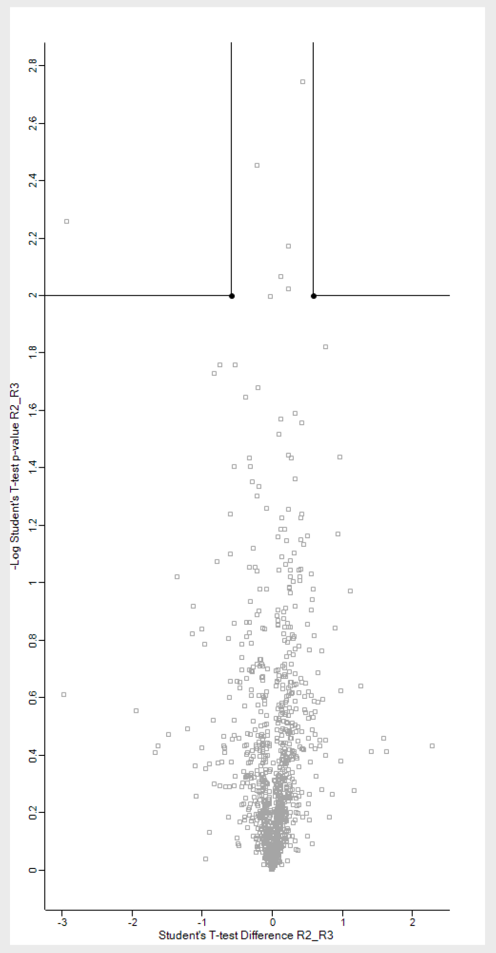

rep 2 vs rep 4

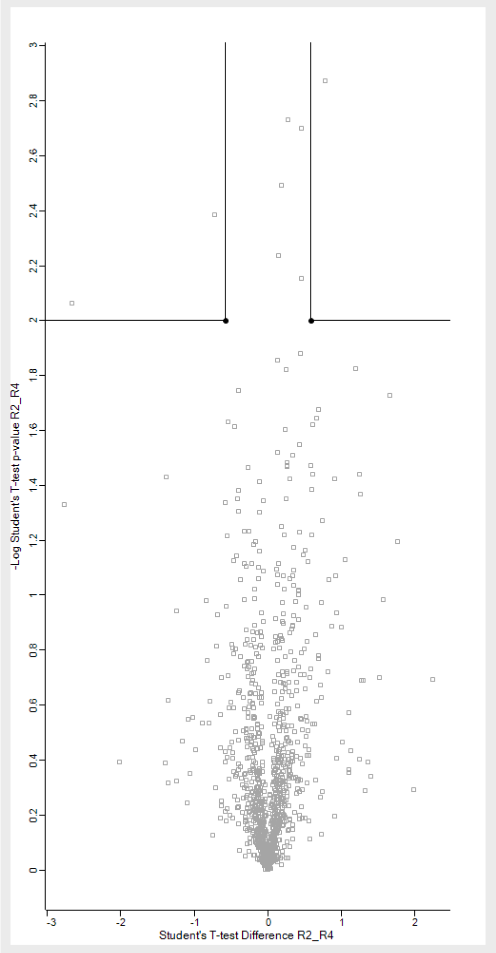

rep 3 vs rep 4

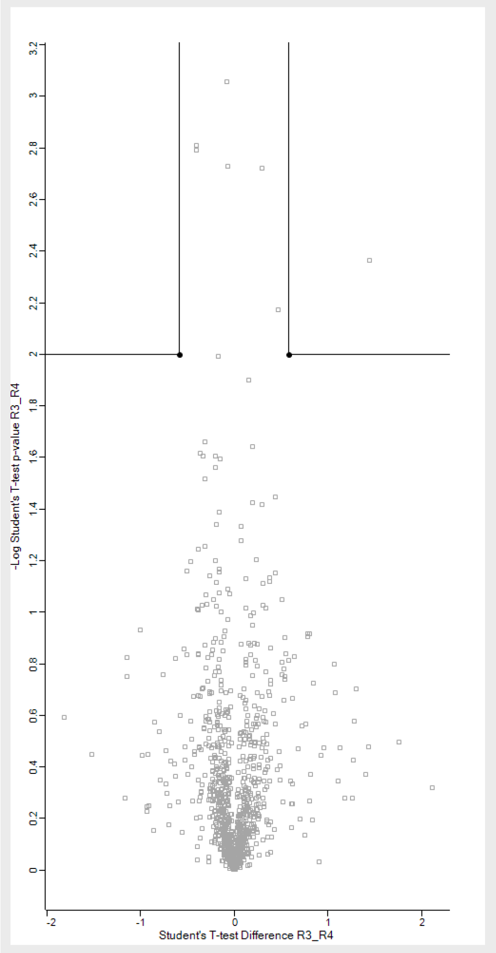

Figure S2: Scatter plots of CTAB samples

stress vs control | rep 3 vs rep 1 | rep 4 vs rep 1 | rep 4 vs rep 3

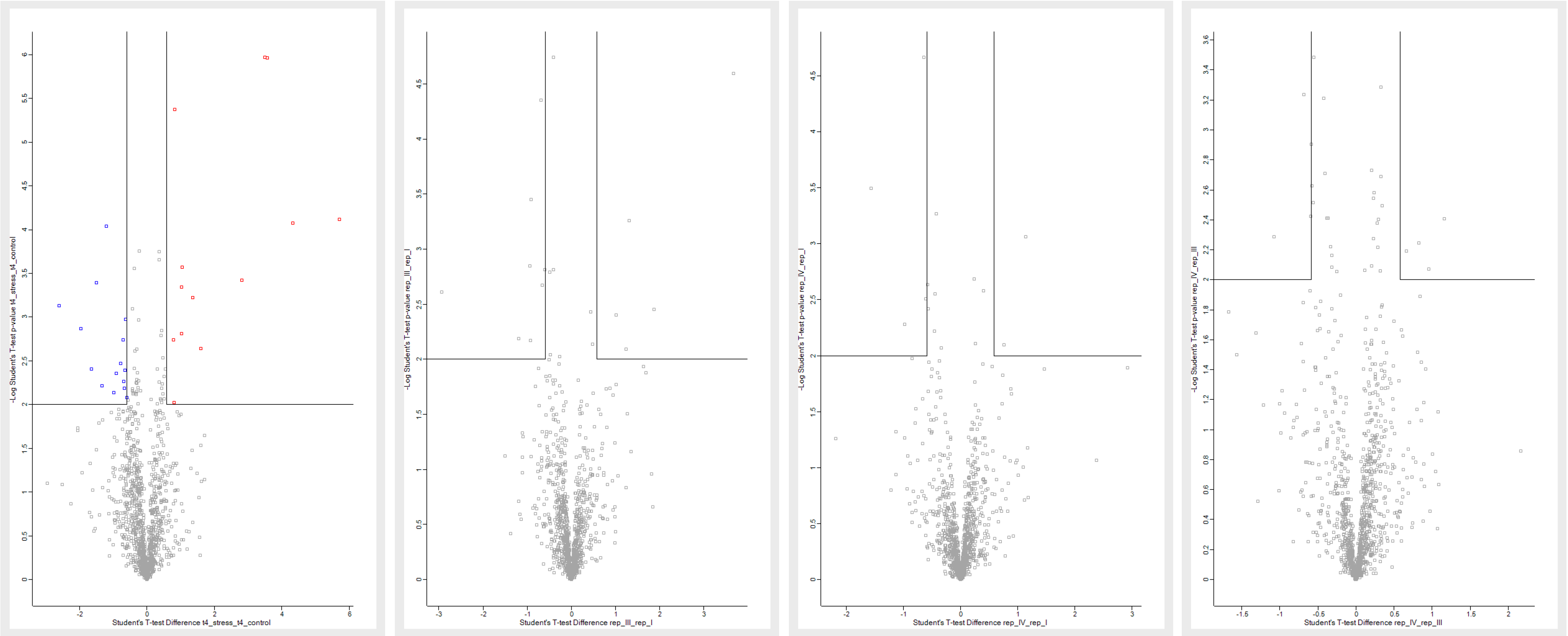

Note that on/off proteins are not shown as significantly changed proteins as no valid fold change and p-value can be assigned to them.
